# Disulfide stress and its role in cardiovascular diseases

**DOI:** 10.1016/j.redox.2024.103297

**Published:** 2024-08-03

**Authors:** Shaoju Qian, Guanyu Chen, Ruixue Li, Yinghua Ma, Lin Pan, Xiaoping Wang, Xianwei Wang

**Affiliations:** a School of Basic Medical Sciences, Xinxiang Medical University, Xinxiang, 453003, China; b Department of Otolaryngology, The First Affiliated Hospital of Xinxiang Medical University, Xinxiang, 453003, China; c Xinxiang Key Laboratory of Tumor Vaccine and Immunotherapy, School of Basic Medical Sciences, Xinxiang Medical University, Xinxiang, 453003, China; d Xinxiang Engineering Technology Research Center of Immune Checkpoint Drug for Liver-Intestinal Tumors, Henan, 453003, China; e Henan Key Laboratory of Medical Tissue Regeneration, Xinxiang Medical University, Xinxiang, China; f Department of Human Anatomy and Histoembryology, Xinxiang Medical University, Xinxiang, China

**Keywords:** Disulfide stress, Cardiovascular diseases, Glutathionylation, Glutaredoxin, Thioredoxin

## Abstract

Cardiovascular disease (CVD) is one of the leading causes of mortality in humans, and oxidative stress plays a pivotal role in disease progression. This phenomenon typically arises from weakening of the cellular antioxidant system or excessive accumulation of peroxides. This review focuses on a specialized form of oxidative stress—disulfide stress—which is triggered by an imbalance in the glutaredoxin and thioredoxin antioxidant systems within the cell, leading to the accumulation of disulfide bonds. The genesis of disulfide stress is usually induced by extrinsic pathological factors that disrupt the thiol-dependent antioxidant system, manifesting as sustained glutathionylation of proteins, formation of abnormal intermolecular disulfide bonds between cysteine-rich proteins, or irreversible oxidation of thiol groups to sulfenic and sulfonic acids. Disulfide stress not only precipitates the collapse of the antioxidant system and the accumulation of reactive oxygen species, exacerbating oxidative stress, but may also initiate cellular inflammation, autophagy, and apoptosis through a cascade of signaling pathways. Furthermore, this review explores the detrimental effects of disulfide stress on the progression of various CVDs including atherosclerosis, hypertension, myocardial ischemia-reperfusion injury, diabetic cardiomyopathy, cardiac hypertrophy, and heart failure. This review also proposes several potential therapeutic avenues to improve the future treatment of CVDs.

## Highlights

• Imbalance in the Grx or Trx system induces disulfide stress.
• Disulfide stress is involved in the occurrence and development of CVDs.
• Disulfide stress is a potential therapeutic target in cardiovascular diseases.

## Graphical abstract

**Figure:**
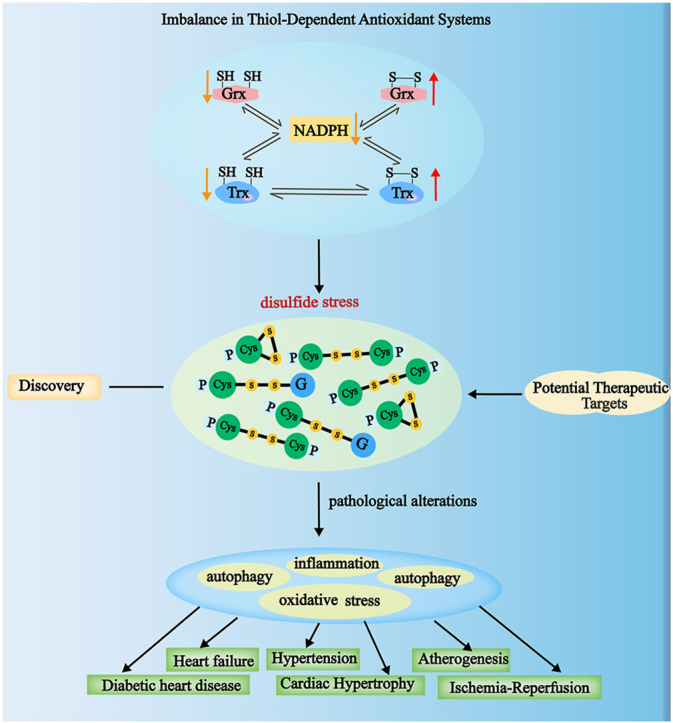

## Abbreviations

AP-1: activator protein 1
AMPK: adenosine monophosphate-activated protein kinase
ASK-1: apoptotic signal-regulating kinase 1
ATM: ataxia telangiectasia
ATP: adenosine triphosphate
BCL-2: B cell/lymphoma-2
cMyBP-C: cardiac myosin-binding protein C
CVD: cardiovascular disease
eNOS: endothelial nitric oxide synthase
ERK: extracellular-signal-regulated kinase
ERO1: endoplasmic reticulum oxidase 1
FOXO3a: forkhead box O3
GAPHD: glyceraldehyde-3-phosphate dehydrogenase
GPX: glutathione peroxidase
Grx: glutaredoxin
GSH: glutathione
GSSG: oxidized glutathione
HDAC4: histone deacetylase 4
HF: heart failure
HO-1: heme oxygenase-1
I/R: ischemia-reperfusion
JNK: Jun N-terminal kinase
MAPK: mitogen-activated protein kinase
MEK: mitogen-activated extracellular signal-regulated kinase
NADPH: Nicotinamide adenine dinucleotide phosphate
Na^+^-K^+^: sodium-potassium
NF-κB: nuclear factor kappa-light-chain-enhancer of activated B
NO: nitric oxide
Nrf2: nuclear factor-erythroid factor 2-related factor 2
PAH: pulmonary arterial hypertension
PASMC: pulmonary arterial smooth muscle cell
PDI: protein disulfide isomerase
PI3K: phosphoinositide 3-kinase
PKB: protein kinase B
PTEN: phosphatase and tensin homolog
RNS: reactive nitrogen species
ROCK: Rho-associated kinases
ROS: reactive oxygen species
SrcFKs: Src family kinases
STZ: streptozotocin
TGFβ: transforming growth factor-β
TRAF2: tumor necrosis factor receptor associated factor 2
Trx: thioredoxin
TrxR: Trx reductase
Trx-S_2_: oxidized Trx
VEGF: vascular endothelial growth factor

## 1 Introduction

Cardiovascular diseases (CVDs) are a major global health challenge, with rising incidence rates owing to an increase in obesity and metabolic disorders. This imposes a significant burden on the socioeconomic and public health systems [1]. Oxidative stress plays a central role in the development of these diseases and contributes to atherosclerosis, myocardial infarction, and heart failure (HF). It also exacerbates damage to the cardiovascular system by affecting cellular signal transduction, gene expression, and cellular metabolism [2,3]. Oxidative stress occurs when there is an imbalance between prooxidant and antioxidant systems, resulting in the accumulation of ROS.

Recent studies have identified a specific type of oxidative stress called disulfide stress which arises from an imbalance in the thiol-dependent antioxidant system, leading to an increase in abnormal disulfide bonds and the dysfunction of sensitive proteases within the cells [4]. Disulfide stress is considered a variant of oxidative stress because it is triggered by an imbalance in the antioxidant system, resulting in increased ROS production and the formation of abnormal intracellular disulfide bonds. The formation of these bonds is related to the accumulation of peroxides. When peroxides increase, cysteine-containing reduced proteins are oxidized to form proteins with disulfide bonds, which help neutralize excessive peroxides and ROS to maintain the ROS balance in the body. The substantial accumulation of proteins with disulfide bonds indicates the presence of high levels of peroxides within the cells [5].

Glutaredoxin (Grx) and thioredoxin (Trx) antioxidant systems are closely associated with disulfide stress and play crucial roles in regulating intracellular redox reactions in the cytoplasm. Disruption of these activities due to certain pathological stimuli triggers the onset of disulfide stress, which is characterized by the sustained glutathionylation of proteins [6], formation of abnormal intermolecular disulfide bonds between cysteine-rich proteins [7], and irreversible oxidation of thiol groups to sulfenic and sulfonic acids [8]. Furthermore, disulfide stress not only increases ROS content, leading to oxidative stress [5], but also activates multiple nuclear factors and protein kinases, resulting in pathological changes such as cellular inflammatory responses, autophagy, and apoptosis.

In this comprehensive review, we investigated the role of disulfide stress triggered by the dysregulation of the thiol-dependent antioxidant system in CVDs. When the Grx and Trx antioxidant systems are imbalanced, disulfide stress is induced, which promotes the production of ROS in cardiomyocytes. Concurrently, the activity of antioxidant enzymes is diminished by the intracellular oxidative environment, leading to oxidative stress and subsequent damage to the cardiomyocytes. Oxidative stress is closely associated with atherosclerosis progression [9]. Furthermore, under hypertension or hypoxia, disulfide stress can induce apoptosis or necrosis in cardiomyocytes or endothelial cells through a cascade of signaling pathways, exacerbating ischemia-reperfusion (I/R) injury [10]. In the decompensated phase of cardiac hypertrophy, disulfide stress increases the oxidative activity of actin, promoting a sustained increase in ventricular wall tension [11]. In the terminal phase, it can further promote glutathionylation of the Na^+^-K^+^ pump, reducing the energy supply to cardiomyocytes and thereby intensifying HF [12].

Glutathione (GSH), the most abundant antioxidant within cardiomyocytes, plays a crucial role in protecting cardiomyocytes from ROS or reactive nitrogen species (RNS) damage through its protein glutathionylation, while the sustained glutathionylation that leads to disulfide stress is a significant factor in exacerbating cardiovascular damage. Grx reduces glutathionylated proteins and restores their activity during the redox signaling cycle. Furthermore, the Trx antioxidant system, particularly Trx1, plays a key role in regulating the redox balance in the cardiac system and is closely related to the development of CVDs. Trx1 responds to disulfide stress signals by modulating nuclear factors such as apoptotic signal-regulating kinase 1 (ASK-1) [13], activator protein 1 (AP-1) [14], phosphatase and tensin homolog (PTEN) [15], and RNA interactions, as well as protein kinase pathways, including Jun N-terminal kinase (JNK), mitogen-activated protein kinase (MAPK), and protein kinase B (PKB), to protect the heart from injury [16]. Therefore, targeting the modulation of Grx and Trx activities to mitigate the damage caused by disulfide stress shows promise as an effective therapeutic approach for CVDs.

## 2 Discovery of disulfide stress

Disulfide stress was first identified in bacterial experiments that detailed the formation of unintended disulfide bonds in cytoplasmic proteins. These formations are caused by environmental oxidative stress or deficiencies in the antioxidant system, which adversely affect protein function and cellular integrity [17,18]. Studies involving the unicellular fungus *Saccharomyces cerevisiae* have reported that elemental sulfur interacts with GSH in the cytoplasm to form glutathione persulfide, hydrogen sulfide, and glutathione disulfide [19]. The accumulation of glutathione disulfide initiates disulfide bond stress and increases ROS levels, which inhibit the growth and development of *Saccharomyces cerevisiae*. This process is similar to sustained intracellular glutathionylation, which leads to extensive clustering of disulfide bond-containing proteins, depletion of reducing equivalents, and induction of thiol protein oxidation. This reduces the cellular capacity to scavenge ROS and triggers disulfide stress. In a rat model of acute pancreatitis, significant increases in disulfide bonds were found between cysteine residues, between γ-glutamyl and cysteine, and between homocysteine molecules in the pancreas. This finding suggests that proteins containing cysteine residues are linked via intermolecular disulfide bonds [4]. Moreover, neuronal studies have reported that the formation of disulfide bonds through the oxidation of neighboring protein thiols also triggers intracellular disulfide stress [20].

An increasing number of studies are reporting that disulfide stress is closely associated with the intracellular thiol-dependent antioxidant system. A persistent decrease in Grx or Trx activity owing to pathological factors can disrupt intracellular redox homeostasis, leading to extensive intermolecular disulfide bonding among cysteine-rich proteins, or the oxidation of thiol groups into irreversible modifications such as sulfinic and sulfonic acids [8,21]. These alterations result in the loss of activity of antioxidant enzymes, such as catalase [22] and glutathione reductase [23], a decline in intracellular antioxidant capabilities, and an increase in ROS, thereby inducing disulfide stress. Additionally, if the activity of cellular regulatory factors, such as nuclear factor-erythroid factor 2-related factor 2 (Nrf2) and nuclear factor kappa-light-chain-enhancer of activated B (NF-κB), is altered due to disulfide bond formation, this may further regulate apoptosis and autophagy, exacerbating disease progression [[24], [25], [26]]. Consequently, disulfide stress, as a distinct form of oxidative stress, is intricately related to an imbalance in the homeostasis of the Trx and Grx systems.

## 3 Imbalance in thiol-dependent antioxidant systems and the triggering of disulfide stress

### 3.1 The role of the Grx and Trx Systems in maintaining redox balance

The Grx and Trx systems play pivotal roles in maintaining redox balance within the cytoplasm by regulating the formation and reduction of disulfide bonds, thus preserving intracellular redox homeostasis. The Grx system consists of GSH, Grx, and glutathione peroxidase (GPX) [6]. GSH, a tripeptide composed of glycine, cysteine, and glutamate, is noteworthy for its ubiquity and multifunctionality in various life forms. It engages in glutathionylation, in which cysteine residues in GSH form disulfide bonds with cysteine residues in target proteins. This process modulates protein activity, participates in cellular signaling, and protects against oxidative damage [21,27]. For instance, the glutathionylation of F-actin reduces actomyosin adenosine triphosphate (ATP)ase activity, thereby affecting cellular contractility [28]. However, chronic glutathionylation results in abnormal disulfide bond accumulation, potentially exacerbating disease progression through intracellular disulfide stress. While Grx reduces glutathionylated proteins and restores their activity in redox signaling cycles, it is a member of the Trx superfamily, thereby resembling the Trx N-terminal structural domain but exhibiting distinct catalytic functions [29]. It is categorized into two main classes: the Grx class, which functions primarily as a disulfide-bond oxidoreductase, and the Grx-like protein class, which serves as an iron sensor and is crucial for transporting iron-sulfur clusters [30].

The Trx system consists of Trx, Trx reductase (TrxR), and nicotinamide adenine dinucleotide phosphate (NADPH). Trx1 operates in the cytoplasm, whereas Trx2 functions in the mitochondria. Trx1 contains five cysteine residues compared to the two cysteine residues at the active site of Trx2, making Trx1 generally more reductive [31]. The Grx and Trx systems collaboratively mitigate oxidative stress by modulating the reduction of disulfide bonds and scavenging ROS through the reducing equivalents provided by NADPH. Together, these systems enhance cellular defense and maintain redox homeostasis [[32], [33], [34]].

**Fig. 1:**
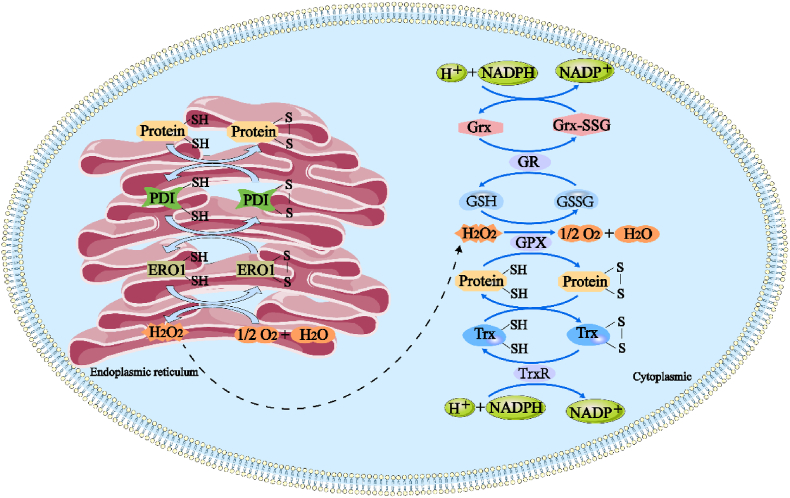
**Grx and Trx systems maintain intracellular redox homeostasis by scavenging excess peroxide through disulfide bond generation and reduction.** Protein disulfide isomerase (PDI) receives electrons from protein folding substrates in the endoplasmic reticulum and oxidizes thiol groups in cysteine residues to form disulfide bonds. ERO1 catalyzes the transfer of electrons from PDI to molecular oxygen using FAD cofactor enzymes, increasing intracellular ROS. The cytoplasmic thioredoxin system, including Trx and thioredoxin reductase (TrxR), participates in the redox process and scavenges ROS by transferring electrons through NADPH. The Grx system, on the other hand, reduces harmful peroxides such as H_2_O_2_ to nontoxic compounds using the catalytic action of glutathione peroxidase (GPX) with GSH. Simultaneously, GSH is oxidized to oxidized glutathione (GSSG), which is then reduced back to GSH by glutathione reductase Grx. Grx is oxidized to form oxidized glutathione-oxidizing protein (Grx-GSSG), which is ultimately reverted back to Grx by the reducing action of NADPH, allowing it to continue participating in antioxidant processes.

Within the cell, one of the main roles of the Grx and Trx systems is to scavenge ROS generated in the endoplasmic reticulum. The specific mechanisms of action are shown in Fig. 1. Under normal physiological conditions, disulfide bond formation is suppressed by high concentrations of reduced GSH in the cytoplasm. Conversely, the oxidizing environment in the endoplasmic reticulum, which is characterized by a high concentration of oxidized glutathione (GSSG), facilitates disulfide bond formation [35]. Disulfide bond formation in the endoplasmic reticulum is primarily orchestrated by the protein-folding coenzymes, endoplasmic reticulum oxidase 1 (ERO1) and protein disulfide isomerase (PDI). PDI catalyzes the oxidation of cysteine residues in proteins to form disulfide bonds, while ERO1 promotes the production of ROS in the endoplasmic reticulum through flavin adenine dinucleotide cofactor-mediated electron transfer, thereby aiding proper protein folding [5,36]. This mechanism accounts for 25 % of the total cellular ROS production and is particularly crucial in cells with high secretory activity [37].

### 3.2 Imbalance in the Grx System and disulfide stress

Glutathionylation, the formation of mixed disulfide bonds between proteins and GSH, is a crucial redox regulatory mechanism that may occur spontaneously or be catalyzed by enzymes. Under normal physiological conditions, glutathionylation protects protein cysteine residues from peroxidation, thereby preserving their structural integrity and functional capacity. It also influences cell signaling as well as cell growth and differentiation. However, under pathological conditions (infection, trauma, radiation, and toxin), excessive glutathionylation can reduce the activity of antioxidant enzymes, including catalase and GPX, thereby weakening the cellular antioxidant defenses and promoting the onset of intracellular disulfide stress [27]. In the mitochondria, excessive glutathionylation can impair the electron transport chain, diminish energy production, and serve as a maladaptive response to oxidative stress [38].

The escalation of oxidative stress can in turn enhance glutathionylation, further exacerbating cellular damage. The GSH/Grx system plays a vital role in maintaining redox homeostasis by regulating protein glutathionylation and deglutathionylation, which are crucial for preserving protein stability and the functionality of antioxidant enzymes. Dysregulation of this system can disrupt intracellular signaling and the redox balance, leading to disulfide stress. For example, in the cardiovascular system, glutathionylation of endothelial nitric oxide synthase (eNOS) influences vasodilation and regulates blood pressure. Increased glutathionylation of eNOS in response to specific stimuli diminishes eNOS activity and adversely affects cardiovascular function. An imbalance in the Grx system can hinder the effective deglutathionylation of eNOS, intensifying disulfide stress in cardiomyocytes and exacerbating myocardial ischemia [39].

**Fig. 2:**
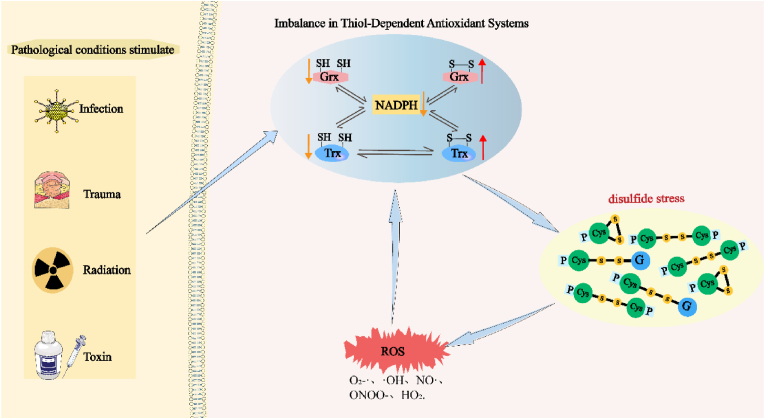
**Imbalance in the Grx or Trx system induces disulfide stress.** Stimulation of external pathological conditions led to an imbalance in the intracellular Grx or Trx system, excessive depletion of NADPH, and a concomitant dramatic increase in oxidative Grx and Trx content, which induced the onset of disulfide stress, the accumulation of a large number of aberrant intracellular disulfide bonds, which then triggered the disruption of the intracellular redox homeostasis and a massive buildup of ROS, which further exacerbated the intracellular disulfide stress and cellular damage.

Reduced GSH levels are associated with neurodegeneration in Parkinson's disease. Lower GSH levels compromise cellular defenses against oxidative stress, leading to enhanced oxidative damage and death of dopamine neurons. Inadequate Grx activity exacerbates this situation by inefficiently clearing glutathionylation, thereby exacerbating disulfide stress and accelerating disease progression [40]. Consequently, under pathological conditions (infection, trauma, radiation, and toxin), decreased or dysregulated Grx activity disrupts the redox balance, increases protein glutathionylation, and facilitates abnormal inter-protein disulfide bond formation, leading to exacerbated disulfide stress and potentially elevated levels of oxidized GSH, thus worsening oxidative stress (Fig. 2).

### 3.3 Imbalance in the trx system and disulfide stress

The Trx system plays a pivotal role in maintaining the redox balance because of its unique activity involving thiol groups, which are essential for cellular vitality. Trx regulates the catalytic activity and substrate-binding sites of target proteins by reducing cysteine residues, thus decreasing the activity of the pro-apoptotic protein, caspase-9, while simultaneously enhancing the activity of the anti-apoptotic protein, B cell/lymphoma-2 (BCL-2) [41]. Furthermore, Trx modulates the interactions between key proteins, such as apoptosis signal-regulating kinase 1 (ASK1)-tumor necrosis factor receptor associated factor 2 (TRAF2) and Bcl-2-Bcl-2 antagonist X (Bcl-2-Bax) [41,42]. It influences the cellular response to disulfide stress by regulating the cysteine residues involved in oligomerization or heterodimerization, thereby mitigating disease onset.

The reduction in Trx activity or expression dysregulation has varied effects on cells depending on the cellular environment (infection, trauma, radiation, and toxin) and characteristics of the target proteins (Fig. 2). For instance, Trx deficiency can lead to increased disulfide stress and DNA damage, exacerbating the formation of disulfide bonds in glyceraldehyde-3-phosphate dehydrogenase (GAPDH), thereby intensifying retinal cell damage [43]. Decreased Trx levels may also inhibit the nuclear localization and transcriptional activity of forkhead box O3a (FOXO3a), reduce the expression of antioxidant enzymes, activate p53, and trigger aging [44]. The reduction or absence of Trx expression weakens the ability of cells to counteract oxidative stress and, in severe cases, can even lead to embryonic lethality [45].

During disulfide stress, oxidized Trx (Trx-S_2_) forms Trx-(SH)_2_, which diminishes the capacity of Trx to reduce target proteins. TrxR, which serves as an electron donor, uses NADPH to facilitate the regeneration of oxidized Trx [22]. However, under high oxidative stress, NADPH may be rapidly depleted by antioxidant enzymes such as GSH peroxidase and TrxR, which are utilized to reduce GSSG and Trx-S_2_. Disulfide stress can also inhibit TrxR activity through alterations in cysteine residues or formation of mixed disulfide bonds. This disrupts the balance between Trx-(SH)_2_ and Trx-S_2_, alters the cellular redox state, exacerbates ROS accumulation, and intensifies disulfide stress. Elevated ROS levels can activate transcription factors, such as Nrf2, NF-κB, and AP-1, thereby regulating Trx gene expression [[24], [25], [26]]. The oxidative intracellular environment, which leads to the oxidation of Trx proteins, further reduces their activity and availability, exacerbating disulfide stress and cellular damage.

## 4 Disulfide stress induces cellular pathological alterations in CVDs

Current research indicates that disruption of the homeostasis of the thiol-dependent antioxidant system leads to disulfide stress, which not only increases ROS content and triggers oxidative stress, but also activates multiple nuclear factors and protein kinases, inducing inflammatory responses, autophagy, and apoptosis, among other pathological changes in cells. As previously mentioned, in the initial stages, the formation of disulfide bonds in intracellular proteins is a protective mechanism that aids in the restoration of the normal physiological state. For instance, in Grx knockout mice, an increase in the adducts formed by hypoxia-inducible factor 1α and GSH in ischemic muscles following femoral artery ligation promotes an increase in vascular endothelial growth factor (VEGF) expression and angiogenesis [46]. Concurrently, the normal formation of disulfide bonds, which protect proteins, may diminish the function of related proteins in the early stages of the disease. In the early stages of the inflammatory response, glutathionylation of inhibitory kappa B kinase β leads to its inactivation, thereby suppressing NF-κB activity and inhibiting the onset of the inflammatory response [47]. However, as the disease progresses and intracellular ROS increases, NF-κB is activated, promoting the secretion of inflammatory factors such as interleukin-6 and TNF-α, exacerbating cellular damage. This suggests that the protective role of disulfide stress may transform into a factor that promotes pathological changes as the disease progresses.

The role of disulfide stress in inducing cellular oxidative stress is multifaceted. Disulfide stress leads to a reduction in the content of reduced Trx and a decrease in its binding to PTEN, thereby enhancing PTEN phosphatase activity, which in turn inhibits phosphoinositide 3-kinase (PI3K)/PKB signaling and promotes ROS production [48]. During the I/R period, disulfide stress can also lead to the glutathionylation of GAPDH, interrupting the energy-producing process of mitochondria and causing ROS accumulation [49]. FOXO3a is a transcription factor that regulates the expression of genes involved in oxidative stress response, DNA repair, apoptosis, and senescence. The activation of FOXO3a depends on the reduction in Trx, which inhibits its ability to be phosphorylated by PKB at its cysteine residues [50]. When disulfide stress occurs, the activity of FOXO3a is reduced, leading to the decreased expression of antioxidant enzymes and increased oxidative stress responses. These studies provide evidence of the crucial role of disulfide stress in oxidative stress; however, there are limitations, such as a lack of an in-depth understanding of the role of disulfide stress in different cell types.

Disulfide stress plays a multifaceted and pivotal role in cellular signal transduction. Upon the onset of disulfide stress, Trx transitions from its reduced to its oxidized form, dissociating from ASK1. Subsequently, the recruitment of TRAF2 and TRAF6 to ASK1 induces autophosphorylation of a critical threonine residue within ASK1, initiating a cascade through the JNK and p38 pathways that leads to apoptosis. This process not only illustrates the direct link between oxidative stress and programmed cell death but also offers potential avenues for the development of therapeutic strategies targeting this pathway. As an oncogenic protein, Ras regulates multiple downstream pathways that influence cell proliferation, survival, migration, and resistance to apoptosis. Disulfide stress can induce mutations in the Ras gene and constitutively activate the Ras protein, further activating the mitogen-activated extracellular signal-regulated kinase (MEK)/extracellular-signal-regulated kinase (ERK) pathway and promoting uncontrolled cell growth and division [51]. Ataxia telangiectasia (ATM), a key regulator of the response to DNA damage, is phosphorylated and activated after DNA damage, initiating multiple downstream targets including the p53/p21 pathway. Disulfide stress exacerbates ATM phosphorylation, impedes its activation, and exacerbates DNA damage [52]. This indicates that the critical role of ATM in the DNA damage response is affected by disulfide stress, which may have significant implications for cancer therapy.

Disulfide stress, an intracellular oxidative state, significantly affects the physiological and pathological conditions of cells through various mechanisms that affect cytoskeletal proteins, signal transduction pathways, angiogenesis, and the function of transcription factors. Disulfide stress also leads to a decrease in the polymerization efficiency of the cytoskeletal proteins, G-actin and F-actin, and a weakening of their binding to myosin, affecting the contractile capacity of the myocardium during I/R [53], offering a new perspective for the treatment of heart disease. Additionally, disulfide stress causes the inactivation of low-molecular-weight protein tyrosine phosphatase through excessive glutathionylation, inhibiting angiogenesis and migration mediated by vascular endothelial growth factor [54]. Furthermore, disulfide stress induces the formation of abnormal intermolecular disulfide bonds in the transcription factor signal transducer and activator of transcription 3, preventing its phosphorylation and thus inhibiting the progression of normal physiological activities, revealing the potential role of disulfide stress in tumor development and other pathological processes [55].

**Fig. 3:**
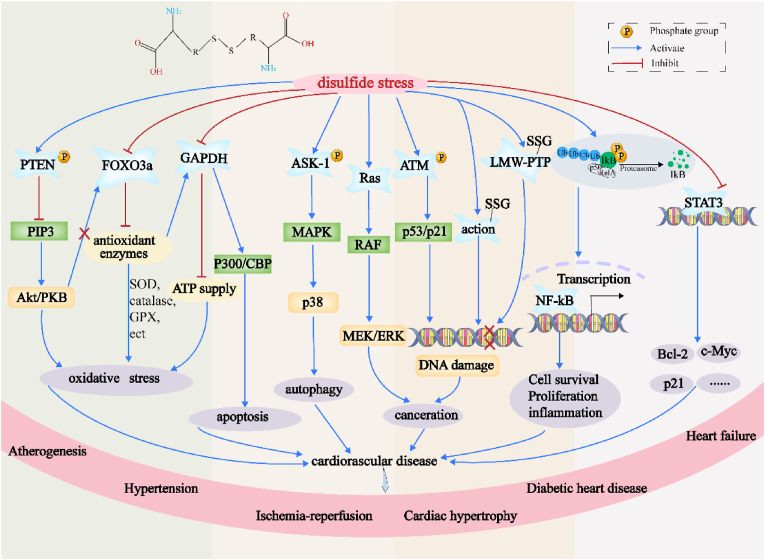
**Illustrates the pathological alterations induced by disulfide stress within cells.** Disulfide stress can activate NF-κB, thus enhancing the secretion of inflammatory cytokines such as IL-6 and tumor necrosis factor-alpha, which exacerbates cellular inflammatory responses. Moreover, disulfide stress diminishes the reductive capacity of thioredoxin, thereby augmenting the phosphatase activity of PTEN and inhibiting the PI3K/PKB signaling pathway, leading to an increase in reactive oxygen species. It also inhibits the activation of FOXO3a and elevates the glutathionylation of GAPDH, promoting oxidative stress. Additionally, disulfide stress, by reducing the reductive thioredoxin, activates the phosphorylation of ASK1, which in turn triggers cellular apoptosis through the JNK and p38 pathways. This stress can also induce mutations in the Ras gene, further activating the MEK/ERK pathway, leading to cellular transformation into cancer. It also aggregates ATM phosphorylation and activates pathways including p53/p21 to promote DNA damage. Furthermore, disulfide stress exacerbates the glutathionylation of actin and LMW-PTP, which respectively inhibit myocardial contractility and angiogenesis. Lastly, disulfide stress also prevents the phosphorylation of STAT3, thereby inhibiting normal physiological activities. The occurrence of all of these mechanisms promotes cardiovascular damage.

In summary, disulfide stress induces oxidative stress within the cell through a variety of mechanisms, leading to pathological changes such as DNA damage and inhibition of angiogenesis via multiple signaling pathways. Severe disulfide stress can also promote cellular autophagy, apoptosis, and carcinogenesis, thereby altering cellular processes (Fig. 3). As a manifestation of oxidative stress, the role of disulfide stress in cellular physiology and pathology warrants further investigation. Future research should focus on the specific roles of disulfide stress at different stages of the disease and explore the development of novel therapeutic strategies by modulating disulfide stress. Further clinical studies are needed to validate the findings from animal models and *in vitro* experiments to ensure the applicability and efficacy of this research.

## 5 The role of disulfide stress in CVDs

CVDs contribute significantly to morbidity and mortality in elderly individuals. CVD encompasses various conditions affecting the heart and blood vessels, including atherosclerosis, hypertension, myocardial I/R injury, cardiac hypertrophy, diabetic heart disease, and HF. Inflammation and oxidative stress are the primary instigators of CVDs [56]. Oxidative stress is associated with detrimental effects such as endothelial dysfunction, vascular smooth muscle cell proliferation, platelet aggregation, thrombosis, fibrosis, apoptosis, necrosis, and autophagy activation [[57], [58], [59]].

This discourse focuses on the role of disulfide stress, triggered by dysregulation of the thiol-dependent antioxidant system, in CVDs. The Trx antioxidant system, particularly Trx1, plays a crucial role in modulating redox balance within the cardiac system and is closely associated with the progression of CVDs. Trx1 responds to disulfide stress signals by regulating nuclear factors, such as ASK-1, AP-1, PTEN, and RNA interactions, as well as protein kinase pathways, including JNK, MAPK, and PKB, thereby protecting the heart from injury [16]. Additionally, GSH, the most abundant antioxidant in cardiomyocytes, is integral to safeguarding these cells from damage caused by ROS or RNS [10]. However, prolonged glutathionylation is a critical factor that exacerbates cardiovascular damage. Persistent glutathionylation diminishes the activity of antioxidant enzymes, activates disulfide stress and related signaling pathways, and affects cellular energy provision and angiogenesis, thus causing damage to myocardial and endothelial cells. Grx reduces glutathionylated proteins, thereby restoring their activity within the redox signaling cycle. Consequently, an imbalance in the Trx and Grx systems is closely associated with the development of CVDs.

### 5.1 Disulfide stress in atherogenesis

Atherosclerosis is a major CVD characterized by intricate immune-inflammatory responses and intracellular oxidative stress [60]. Disulfide stress plays a significant role in the pathogenesis of atherosclerosis. An early hallmark of atherosclerosis is increased vascular permeability, which is closely associated with the impairment of endothelial cell barrier function. The maintenance of vascular permeability relies on the integrity of the endothelial cell barrier, which is strictly controlled by actin cytoskeleton dynamics [61]. Specifically, persistent glutathionylation of actin at Cys 81 and Cys 157 by Rac 1 diminishes its activity, leading to the loss of cortical actin structure, an increase in stress fibers, and disassembly of intercellular adhesion junctions, thereby altering the cytoskeletal structure and integrity of cell-to-cell adhesion. This plays a pathogenic role in the metabolic stress-induced dysfunction of the aortic barrier [62]. Experiments have revealed that overexpression of Grx in aortic endothelial cells can remove the glutathionylation of Rac 1, restore its activity, stabilize the actin cytoskeleton structure, protect barrier integrity, and ameliorate endothelial barrier dysfunction under metabolic stress conditions [63]. Grx overexpression not only improves endothelial barrier dysfunction but also mitigates the phenotype of hyperlipidemia by reducing the acetylation of Srebp1. Grx deficiency leads to irreversible glutathionylation of Silent Information Regulator 1(SIRT1), accelerating disulfide stress in mice fed a high-fat diet, owing to the accumulation of SIRT1 protein intermolecular disulfide bonds. Supplementation with adenoviral Grx can restore SIRT1 activity, reduce the acetylation of Srebp1, and ameliorate the hyperlipidemic phenotype [64]. These findings suggest that Grx is a useful target for the treatment of atherosclerosis. However, these outcomes require further validation in clinical and physiological settings to ensure their universality and efficacy. Additionally, endothelial cells resist disulfide stress through protective mechanisms that prevent apoptosis. The luminal flow of blood also upregulates Grx activity, reducing the expression of Bim through the PKB-FoxO 1 signaling pathway and inhibiting JNK activation of Bim, thus preventing endothelial cell apoptosis [65]. Grx, a potential regulatory factor, warrants further investigation for its potential to reduce disulfide stress and improve endothelial dysfunction. Further research is needed to understand the interplay between disulfide stress and other cardiovascular risk factors and how these interactions affect the onset and progression of the disease. Such studies can improve our understanding of the complexity of atherosclerosis and provide a scientific basis for the development of new therapeutic strategies.

Current research indicates that the Trx system plays a protective role against atherosclerosis by mitigating the effects of disulfide stress. The expression levels of Trx protein and mRNA in endothelial and macrophage cells within atherosclerotic plaques are elevated, and serum Trx levels are higher in patients with active variant angina than those in patients in the inactive phase, suggesting an antioxidant role of Trx in atherosclerosis [66]. Furthermore, the Trx antioxidant system modulates inflammatory manifestations by binding to target proteins via disulfide bonds. However, subsequent studies revealed that in macrophages under endotoxic attack, Trx-80 significantly enhances the differentiation of inflammatory M1 macrophages, indicating the potential complexity of the role of the Trx system in atherosclerosis. Trx-1, by downregulating the expression of p16INK4a, enhances the expression of anti-inflammatory M2 macrophages and dampens the expression of pro-inflammatory M1 markers, such as TNF-a and MCP-1, by binding to transcription factors, reducing the formation of foam cells [14]. This is in contrast to the role of Trx-80 in promoting the differentiation of inflammatory M1 macrophages into macrophages under endotoxin attack [67]. This contrast underscores the complexity of the role of the Trx system in atherosclerosis and suggests that different Trx subtypes may play distinct roles in disease progression. However, the different mechanisms of action of Trx-1 and Trx-80 in atherosclerosis indicate the potential complexity of their roles in the Trx system. Future research is needed to delve into the specific roles of Trx-1 and Trx-80 in disease progression and their interactions with other cardiovascular risk factors. Further research is also required to assess the potential and safety of the Trx system as a therapeutic target and provide a scientific basis for the development of new treatment strategies.

### 5.2 Disulfide stress in hypertension

Hypertension is the principal risk factor for a multitude of CVDs, including ventricular dysfunction, coronary artery disease, and HF. Among the mechanisms underlying its development, vascular relaxation dysfunction is evident and is closely associated with disulfide stress occurring in endothelial cells. Hypertension can induce alterations in the glutathionylation of eNOS, affecting its capacity to produce nitric oxide (NO). Studies have reported heightened glutathionylation of eNOS in the vasculature of spontaneously hypertensive rats, leading to impaired endothelium-dependent vasorelaxation. Glutathionylated eNOS remains sequestered in the cytoplasm for extended periods, inciting disulfide stress and is potentially degraded through the chaperone-mediated autophagy pathway, resulting in an irreversible loss of eNOS and restricted vasorelaxation [68]. In contrast, normotensive control rats exhibited lower eNOS glutathionylation levels and normal vasorelaxation responses [69]. Moreover, eNOS glutathionylation may represent a single facet of the complex pathophysiological processes in hypertension, and other factors, such as actin glutathionylation, may also play a role in its progression. When subjected to disulfide stress induced by hypertension in cardiomyocytes, actin, a redox-sensitive protein within the body, can lead to the glutathionylation of titin and cardiac myosin-binding protein C (cMyBP-C), further exacerbating vasorelaxation dysfunction and promoting the onset of hypertension [70].

Furthermore, disulfide stress may modulate vascular tone and endothelial cell proliferation by affecting signaling pathways within the vasculature, such as Src family kinases (SrcFKs) and Rho-associated kinases (ROCK). Under hypertensive stimuli, sustained glutathionylation of SrcFKs and ROCK may suppress the proliferation of vascular smooth muscle cells through multiple signaling pathways, promote apoptosis, and decrease vascular tone [71]. Thus, targeting the elimination of disulfide stress caused by persistent glutathionylation to restore vasorelaxation shows promise as an effective method for treating hypertension. Additionally, existing research has determined that Trx can reverse arterial stiffness and improve endothelial function, thereby treating aged hypertensive wild-type (WT) mice and reducing blood pressure to levels comparable to young WT mice, offering a transient therapeutic effect on hypertension. This therapeutic mechanism involved the injection of recombinant Trx into mice, inducing the activation of mitogen-activated protein kinase 4 in endothelial cells, which in turn reduced the activity of the transforming growth factor-β (TGFβ)-matrix metalloproteinase-2- TGFβ receptor II pathway, decreased the aggregation of extracellular matrix protein disulfide bonds, and mitigated the progression of arteriosclerosis, providing a transient therapeutic effect on hypertension [72].

Pulmonary arterial hypertension (PAH) is a severe form of hypertension characterized by increased pulmonary vascular resistance and remodeling, ultimately leading to right ventricular dysfunction and failure [73]. Disulfide stress triggered by dysregulation of the Trx system is considered a significant factor in PAH development. Studies have found that PAH induced by monocrotaline affects the expression of vitamin D-regulated gene-1 and Nrf2, suppressing the expression of Trx-1, thereby inducing disulfide stress and increasing ROS, which in turn inhibits the proliferation of pulmonary arterial smooth muscle cells (PASMCs). Moreover, under both chemical induction and hypoxic conditions, the mRNA and protein levels of Trx2 decrease in human pulmonary artery endothelial cells and human PASMCs, promoting disulfide stress and the development of PAH, which is an adaptation of the body to a hypoxic environment [74]. In a subsequent chronic hypoxia mouse model, the activity of Trx1 in the lungs of adult mice increased, and Trx1 mitigated the impact of disulfide stress on vascular cells by activating HIF and the subsequent PI3K-PKB signaling pathway, promoting hypoxia-induced PASMC proliferation and pulmonary vascular remodeling. Although the protective role of Trx1 in PAH has been confirmed, these studies often do not consider the regulatory mechanisms of Trx1 activity or differences in its expression in different patients with PAH. Future research should pay more attention to the regulatory mechanisms of Trx1 activity and its expression and functional differences in patients with PAH, which will help slow down the development of disulfide stress in PAH and thus help to develop effective treatment strategies.

### 5.3 Disulfide stress in diabetic heart disease

Diabetic cardiomyopathy is a prevalent complication among patients with diabetes and is characterized by the onset of myocardial dysfunction in the absence of other overt cardiovascular risk factors [74]. With the increasing incidence of diabetes, a deeper understanding of the pathogenesis of diabetic cardiomyopathy is important for its prevention and treatment. Early features of the disease include myocardial fibrosis, functional impairment, and diastolic dysfunction, which may progress to systolic dysfunction and ultimately lead to clinical HF [75]. Hyperglycemia induced by diabetes triggers an imbalance in the Grx antioxidant system, leading to disulfide stress and an increase in ROS, which play pivotal roles in the development of diabetic cardiomyopathy. In endothelial cells isolated from patients with type 2 diabetes, the level of glutathionylated proteins is significantly elevated, revealing the impact of oxidative stress on the cardiovascular system under hyperglycemic conditions [63]. Under conditions of low ROS, the glutathionylation of adenosine monophosphate-activated protein kinase (AMPK)-α mediated by Grx can activate AMPK, improve glucose transport and degradation, and simultaneously inhibit glycogen synthesis while maintaining redox balance. However, in an environment of high ROS concentration, persistent glutathionylation of the AMPK protein induced by ROS leads to disulfide stress, exacerbating diabetic cardiovascular diseases induced by hyperglycemia [76]. Moreover, in hyperglycemia induced by high glucose levels and insulin receptor blockade, glutathionylation of the cardiac Na-K pump can cause functional reduction and disulfide stress, inducing myocardial apoptosis. These findings suggest that Grx plays different roles in various redox environments, which is crucial for understanding the complex pathological processes of diabetic cardiomyopathy.

Targeting the prevention and treatment of persistent glutathionylation of intracellular proteins to combat disulfide stress in diabetes is a viable approach. For example, GSH can prevent β-cell failure and pre-diabetes induced by chronic oscillating glucose administration. Supplementation with GSH promotes the glutathionylation of Keap1 and the translocation of Nrf2 to the nucleus, enhancing the expression of glutamate cysteine ligase catalytic subunit, Grx1, heme oxygenase-1 (HO-1), and NADPH: quinone acceptor oxidoreductase 1, while reducing the production of ROS. In addition to GSH supplementation, Grx-1 gene therapy protects cardiomyocytes from diabetes [77]. Grx-1 gene therapy mitigates myocardial infarction, apoptosis, and death signals associated with diabetes and I/R via the ASK-1/JNK/p38 MAPK pathway, while enhancing the survival signals of cardioprotective proteins, such as the phosphorylation of Akt and FoxO-1, and the induction of e-NOS and the cardioprotective phase II enzyme, HO-1 [78]. This complements the mechanism of action of GSH supplementation and provides a comprehensive treatment strategy for diabetic CVDs. In coronary artery endothelial cells, Grx1 treatment can protect cells from hyperglycemia-induced disulfide stress and apoptosis and reverse the glutathionylation of eNOS induced by hyperglycemia, restoring its activity and NO levels, further confirming the potential of Grx1 in the treatment of diabetic CVDs [79]. Future research should delve deeper into the applicability and long-term therapeutic effects of these strategies in various states of diabetes and their interactions with other cardiovascular risk factors to provide more scientific evidence for the treatment of diabetic CVDs.

Insulin response and glucose uptake are impaired in patients with type 2 diabetes, leading to hyperglycemia, the most common form of diabetes, which is considered a major risk factor for CVDs [80]. Studies indicate that leakage of the cardiac ryanodine receptor calcium release channel and desynchronization of local calcium release can exacerbate heart function in patients with diabetes [81]. This is associated with a reduction in Trx-1 activity in diabetes, which promotes the occurrence of disulfide stress in cardiomyocytes. Furthermore, in the H9c2 cardiac cell model cultured under *in vitro* hyperglycemic conditions, the reduced activity of Trx-1 may be caused by its inhibition of Trx-1 via the metabolic byproduct, methylglyoxal, which also induces intracellular disulfide stress, activates the p38-MAPK pathway, and reduces the binding of Trx-1 to ASK-1, thereby promoting myocardial cell apoptosis [82]. Diabetes not only reduces Trx1 activity but also inhibits the activity of Trx2 in the mitochondria, triggering disulfide stress. In H9C_2_ cardiac cells treated with high glucose and the myocardium of streptozotocin (STZ)-induced diabetic rats, the expression of Trx2 is reduced, increasing mitochondrial oxidative damage and decreasing ATP production in H9c2 cells [83].

However, in the cardiac mitochondria of Zucker diabetic fatty rats, a diminished presence of ROS and enhanced ROS-scavenging capacity of the GSH/Trx antioxidant system have been observed, suggesting that the intracellular antioxidant system may play a protective role in the early stages of diabetes [84]. Elevating the activity of Trx proteins or overexpressing the Trx gene could be an effective method for preventing diabetic cardiomyopathy. In the diabetic milieu, angiogenesis is often impaired by the downregulation of angiogenic growth factors, and inducing the expression of Trx1 can protect the diabetic myocardium from angiogenic injury by reducing disulfide stress and enhancing the expression of HO-1 and VEGF [84]. Furthermore, adenoviral recombinant gene therapy for Trx1 can significantly increase the expression of Trx1 in the myocardium of STZ-induced diabetic rats, decrease disulfide stress and ROS, alleviate myocardial infarction and fibrosis, increase the density of capillaries and small arteries, reduce infarct area, and improve cardiac function [85,86]. These findings suggest that targeting Trx-1 for the treatment of disulfide stress may be an efficacious therapeutic approach, aiding in the prevention and treatment of cardiac complications in patients with diabetes.

### 5.4 Disulfide stress in myocardial I/R injury

Myocardial I/R is often preceded by ischemic heart disease, and studies have indicated that ischemia can augment protein glutathionylation, potentially triggering intracellular disulfide stress. During I/R, glutathionylation of G-actin diminishes its polymerization rate and binding affinity for myosin, thereby attenuating myocardial contractility in rats during ischemia [87]. Another study revealed that acute hypoxia can induce glutathionylation of Na^+^-K^+^-ATPase on the cardiomyocyte membrane, inhibiting its activity and reducing ATP consumption before the cell transitions to anaerobic glycolysis [88]. These experiments suggest that during the pre-ischemic phase, protein glutathionylation helps maintain a low-energy state in cells, thereby mitigating damage to cardiomyocytes from hypoxia. In the recovery phase of I/R injury, the use of nitrosoglutathione can enhance glutathionylation of Na^+^-K^+^-ATPase, thus accelerating the recovery of contractility in isolated rat myocardium [89].

During myocardial I/R, persistent protein glutathionylation can lead to disulfide stress. Experiments have shown that this process induces glutathionylation of GAPDH in human aortic endothelial cells, reducing its functionality, increasing disulfide stress, decreasing glycolysis, and ultimately leading to increased apoptosis. Grx has the potential to protect the heart by alleviating apoptosis caused by disulfide stress. Experiments have found that mice deficient in Grx1 exhibit more severe myocardial infarction after ischemia, whereas those overexpressing Grx1 show milder I/R injury [10]. Grx3 can shield the heart from I/R damage, with its absence leading to larger myocardial infarctions and its overexpression mitigating myocardial damage. Furthermore, compared to Grx3 knockout mice, the hearts of mice overexpressing Grx3 exhibit reduced AngII-induced ROS release and H_2_O_2_-mediated apoptosis [90]. Thus, during the pre-ischemic phase, intracellular protein disulfide stress can reduce energy expenditure and cellular burden. However, sustained glutathionylation, which leads to disulfide stress, can cause cardiomyocyte death and exacerbate myocardial cell injury during I/R. Although the role of disulfide stress in myocardial injury is widely accepted, studies have often overlooked the interplay between disulfide stress and other cellular stress pathways. Future research should focus on the interaction between disulfide stress and other cellular stress pathways, as well as the specific impact of these interactions on myocardial I/R injury.

Stress-induced hyperglycemia is a prevalent symptom in patients with cardiac diseases following I/R injury and is closely associated with the incidence and mortality of CVDs independent of diabetes [91]. An acute hyperglycemic state significantly augments apoptosis and disulfide stress in cardiomyocytes, and transient exposure to hyperglycemia exacerbates the prognosis of myocardial I/R. Melatonin can enhance Trx activity and downregulate the expression of Trx-interacting protein (Txnip), significantly strengthening the Notch1/Hes1/PKB signaling pathway, rescuing disulfide stress injury caused by the imbalance of the Trx antioxidant system, and effectively reducing the infarct size and the rate of cardiomyocyte apoptosis [92]. Furthermore, the mitochondrial Trx2 antioxidant system also plays an *anti*-disulfide stress role during reperfusion through a variety of signaling pathways. For instance, the delivery of lentiviral vectors encoding green fluorescent protein-trx2 to the myocardium of a rat myocardial I/R injury model markedly increased the expression of Trx2. Its upregulation activates PKB by binding to it, inhibiting abnormal autophagy in the myocardium, alleviating disulfide stress, reducing myocardial histopathological injury, and decreasing myocardial enzyme leakage and infarct size [83]. However, the limitations of these studies lie in the insufficient consideration of the dynamic changes in these molecules at different stages of I/R injury and their interactions with other cellular stress pathways. Future research should delve deeper into the mechanisms of action of these molecules and their cell type-specific roles in myocardial I/R injury.

### 5.5 Disulfide stress in cardiac hypertrophy

Cardiac hypertrophy is commonly associated with various CVDs and typically occurs during the decompensated phase of heart function. It is characterized by an increase in cardiomyocyte size, which may initially confer benefits by reducing ventricular wall tension and enhancing cardiac pump function. However, in the long term, it can significantly increase the risk of HF, sudden death, and other cardiac diseases [93]. Among the myriad causes of cardiac hypertrophy, the Raf/MEK/ERK pathway is particularly important. In hypertrophied hearts, Grx1 exerts anti-proliferative effects by inhibiting the Ras/Raf/Erk pathway [94]. However, when myofibrils in cardiomyocytes are overstretched under mechanical stretch stimuli, the accumulation of intracellular ROS due to various causes leads to persistent glutathionylation at the Cys 118 site of Ras. Disulfide stress increases Ras activity and activates the Raf/MEK/ERK signaling pathway, thereby inducing cardiomyocyte hypertrophy [11]. Overexpression of Grx1 can serve as a response to the sustained glutathionylation of Ras, inhibiting glutathionylation and ERK activation, thereby ameliorating cardiac hypertrophy. Thus, Grx1 suppresses the progression of cardiac hypertrophy by combating disulfide stress. In addition to Grx1, Grx2, and Grx3 play beneficial roles in cardiac hypertrophy. Mice deficient in Grx2 exhibit cardiac hypertrophy, fibrosis, and hypertension. Loss of Grx2 function leads to the dysregulation of mitochondrial protein glutathionylation, particularly a decrease in complex I activity, impaired myocardial mitochondrial function, altered redox status, and reduced ATP output [95]. Addition of dithiothreitol to the mitochondria of Grx2-deficient hearts improves complex I glutathionylation and restores mitochondrial function. This illustrates that reductants targeting disulfide bonds can effectively suppress disulfide stress. Furthermore, one study reported that in Grx3 knockout mice, an increase in disulfide-bonded proteins and ROS production in cardiomyocytes led to significant left ventricular hypertrophy by 12 months of age, resulting in cardiac hypertrophy and hypertension [96]. Therefore, Grx plays a key role in myocardial cell hypertrophy by modulating protein glutathionylation in hypertrophic cells through the antioxidant system, mitigating the damage caused by disulfide stress, and delaying the progression of myocardial hypertrophy.

Moreover, an increasing body of research indicates that Trx1 is an important endogenous negative regulator of cardiac hypertrophy, and the inactivation of Trx1 may be one of the mechanisms underlying myocardial hypertrophy. Under pathological conditions, Trx1 is oxidized and inactivated by the formation of a disulfide bond between Cys-62 and Cys-69 [97]. The mixed disulfide bond formed between Trx1 Cys-73 and glutathione also diminishes Trx1 activity [98]. In addition, ROS may promote the degradation Trx1 via a cathepsin D-dependent mechanism [99]. These post-translational modifications, leading to the inactivation of Trx1, cause direct activation of its interacting molecules, increasing ROS, and the formation of disulfide bonds between cysteine residues in normal cardiomyocytes, thereby causing myocardial hypertrophy. Elucidating these mechanisms will provide a new perspective on the role of Trx1 in cardiac hypertrophy. Furthermore, another study reported that mice with a cardiac-specific gene deletion of Trx1 exhibited increased disulfide stress accompanied by myocardial hypertrophy. In contrast, transgenic mice with cardiac-specific Trx1 overexpression show attenuated responses to pressure overload and reduced levels of disulfide stress. These results emphasize the protective role of Trx1 in cardiac hypertrophy and highlight the complexity of the regulation of Trx1 activity [100]. Both Trx1 and mitochondrial Trx2 play key roles in maintaining the redox status and controlling mitochondrial ROS release, thereby inhibiting disulfide stress. Mice with cardiac-specific deletion of Trx2 exhibit increased synthesis of cell surface proteins, reduced AMPK-α activity, leading to severe early-onset hypertrophy, decreased myocardial contractility, and earlier mortality [101]. This indicates that mitochondrial Trx2 is crucial for cardiac health. However, the specific mechanism of mitochondrial Trx2 action in cardiac hypertrophy requires further investigation.

The Trx system, a principal thiol-dependent antioxidant mechanism, shields the hypertrophied myocardium from disulfide stress injury under various pathological conditions. Under stress conditions such as pressure overload, ischemia, and HF, the upregulation of cytosolic Trx1 expression is considered an endogenous compensatory mechanism against myocardial damage [102]. Studies have indicated that PTEN, a tumor suppressor protein, forms a disulfide bond with Trx1 at its catalytic Cys32 and the PTEN C2 lipid-binding domain cysteine at position 212, diminishing the phosphatase activity of PTEN and activating the PI3K/PKB signaling pathway in NIH3T3 cells, thereby mitigating disulfide stress injury to cardiomyocytes [15]. This discovery is important for understanding the protective mechanisms of Trx1 against cardiac hypertrophy. However, existing research has not fully considered the variations in Trx1 expression and activity under different stress conditions and how these variations affect the progression of cardiac hypertrophy.

Furthermore, ASK-1, a mitogen-activated protein kinase, interacts with reduced Trx1 under normal conditions, inhibiting its kinase activity. Upon external stimuli, increased ROS oxidizes Trx1, leading to its dissociation from ASK-1 and the activation of the downstream P38/JNK pathway, inducing apoptosis and hypertrophy in cardiomyocytes [103]. Thus, under pathological conditions, functional alterations of Trx1 may lead to myocardial cell injury through various mechanisms. In the nucleus, reduced histone deacetylase 4 (HDAC4) plays a key role in the regulation of cardiac hypertrophy by inhibiting the target genes of multiple transcription factors, such as serum response factor, nuclear factor of activated T cells, and myocyte enhancer factor 2. Under hypertrophic stimuli, the disulfide bond formed between Cys-667 and Cys-669 of HDAC4 is oxidized and exported from the nucleus, while Trx-1 reduction of oxidized HDAC4 bound with Txnip and DnaJb5 allows HDAC4 to re-enter the nucleus and exert its anti-hypertrophic effect [104]. This finding emphasizes the dual role of Trx1, both inside and outside the nucleus, in cardiac hypertrophy.

In summary, intracellular disulfide stress may be a crucial mechanism mediating myocardial hypertrophy. Trx1 inhibits disulfide stress in various ways under stress conditions, preventing the progression of cardiac hypertrophy, an important endogenous compensatory mechanism against myocardial injury. Future research needs to delve deeper into these intermolecular mechanisms and their integrated impact on cardiac hypertrophy to provide targets for the treatment of cardiac hypertrophic injury caused by altered Trx activity and disulfide stress.

### 5.6 Disulfide stress in HF

Prolonged adverse adaptations in myocardial cells can lead to severe heart failure and subsequent mortality. HF is a clinical syndrome caused by a variety of etiologies and is one of the leading causes of death in humans. Myocardial hypertrophy results in decreased total phosphorylation of myofilament proteins and impaired contractile function, leading to HF [105]. cMyBP-C) is a thick filament-associated protein involved in the regulation of cardiac contraction and relaxation [106]. In patients with end-stage HF, most of the cMyBP-C Cys 249 subunit undergoes persistent glutathionylation, inhibiting its phosphorylation and affecting its binding to myosin, resulting in impaired myocardial cell contractility [107]. Another study found that glutathionylation of the Na^+^-K^+^ pump inhibits the excitation-contraction coupling activity of myocardial cells, exacerbating HF. Grx1 can reduce the glutathionylation of the Na^+^-K^+^ pump, thereby increasing its inhibitory effect and revealing the potential role of ion pumps in HF [12]. Moreover, growing evidence suggests that redox-sensitive proteins, including Grx, protect cardiomyocytes from disulfide stress-induced injury.

Dilated cardiomyopathy, diabetic cardiomyopathy, and myocardial hypertrophy are common causes of HF, which is an irreversible end-stage manifestation of these diseases. Molecular analysis of the myocardium of patients with dilated cardiomyopathy revealed that disulfide stress in CVD persists at every stage, with mitochondrial ultrastructural damage, increased membrane potential, increased ROS release, increased formation of intermolecular disulfide bonds in intracellular thiol proteins, reduced ATP generation, and increased myocardial cell apoptosis dependent on ASK-1 [108]. Furthermore, in mice with cardiac-specific deletion of Trx1, there is a significant increase in the content of disulfide-bonded proteins caused by disulfide stress compared to the WT, and excess H_2_O_2_ inhibits mammalian target of rapamycin activity by forming intermolecular disulfide bonds at the Cys 1483 site, leading to the downregulation of metabolic gene expression of pS6K and p4EBP1 in cardiomyocytes, mitochondrial dysfunction, and cardiac function failure [109]. This further emphasizes the role of disulfide stress in myocardial cell apoptosis and dysfunction. These findings suggest that disulfide stress may be a key link between different cardiomyopathies and HF. Future research should explore the detailed mechanisms of disulfide stress in the development of HF and assess interventions targeting disulfide stress.

Trx1 overexpression also inhibits mitochondrial dysfunction in septic mice, promotes mitochondrial recovery, prevents HF, and reduces doxorubicin-induced disulfide stress injury to the mitochondria [110]. In a septic mouse model, Trx1 overexpression inhibited mitochondrial dysfunction, promoted mitochondrial restoration, reduced doxorubicin-induced disulfide stress injury to mitochondria, and prevented the onset of HF. Therefore, Trx1 has potential therapeutic value in mitochondrial protection and the prevention of HF. Additionally, in a myocardial infarction rat model with type I diabetes, injection of Trx1 adenovirus reduced the number of disulfide bonds in myocardial cells and improved cardiac function [86]. These results highlight the potential of Trx1 in HF treatment. However, existing research may not have fully considered the expression and activity of Trx1 in different cardiac pathological states and its interaction with other signaling pathways. The synergistic effects of Grx and Trx1 may be a promising strategy for the treatment of HF. Future studies should explore the interactions among these proteins and how they collectively regulate the redox states of cardiomyocytes.

**Fig. 4:**
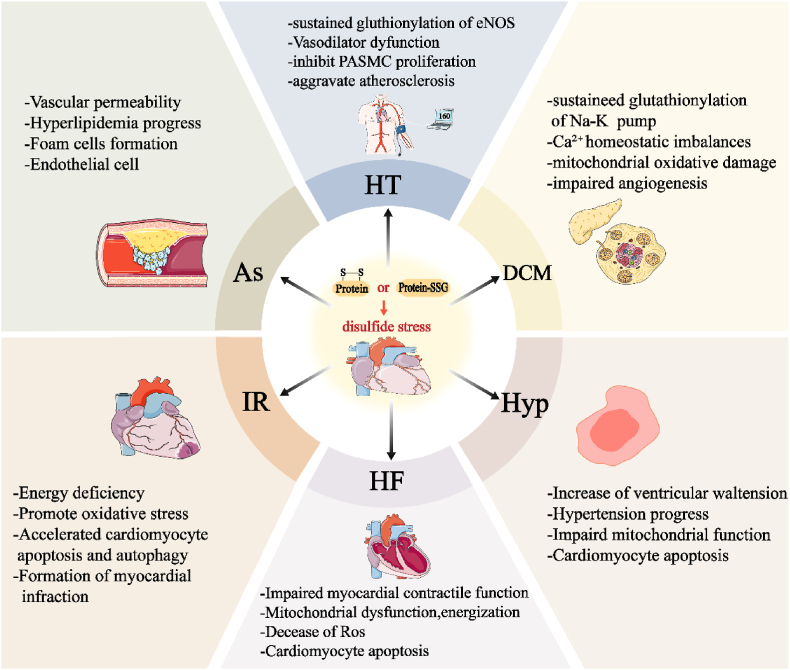
**Specific impairing effects of disulfide stress in cardiovascular diseases.** When the balance of the Grx and Trx Systems is disrupted, triggering disulfide stress, it increases vascular endothelial permeability, accelerates the development of hyperlipidemia, promotes the formation of foam cells and endothelial cell apoptosis, and exacerbates arteriosclerosis. In hypertension, disulfide stress induces sustained glutathionylation of eNOS, vasodilatory dysfunction, inhibition of pulmonary artery smooth muscle cell and endothelial proliferation and increased aggregation of disulfide bonds between cellular matrix proteins, and promotion of cardiovascular cell injury. During myocardial ischemia-reperfusion, disulfide stress leads to the oxidation of biomacromolecules, reduces the blood supply during the ischemic phase, triggers oxidative stress, and accelerates myocardial apoptosis and infarction. In myocardial hypertrophy during the cardiac decompensation phase, disulfide stress increases ventricular wall tension, maintains hypertension, impairs mitochondrial function in myocardial cells, and promotes myocardial apoptosis, exacerbating myocardial hypertrophy. In diabetic heart disease, disulfide stress induced by hyperglycemia promotes sustained glutathionylation of the cardiac Na^+^-K^+^ pump, exacerbates Ca^2+^ homeostatic imbalances, promotes mitochondrial oxidative damage and inhibition of VEGF production, allowing impaired angiogenesis and heart failure. In the later stages of heart disease, when heart failure occurs, disulfide stress further weakens cardiac contractility, causes mitochondrial dysfunction and reduced energy production, leading to ROS accumulation and accelerated myocardial cell apoptosis.

In summary, disulfide stress plays a crucial role in various CVDs, such as atherosclerosis, hypertension, diabetic cardiomyopathy, myocardial I/R, cardiac hypertrophy, and HF(Fig. 4). The Grx and Trx systems are responsible for regulating intracellular redox balance, which greatly influences the progression of these diseases. However, most current research focuses on cellular and animal models, and there is still a need for clinical validation of their therapeutic effectiveness. Future research should explore the variations in the activity of the Grx and Trx systems under different pathological conditions and their interactions with other cardiovascular risk factors. Additionally, large-scale, multicenter clinical studies are necessary to evaluate the long-term efficacy and safety of therapeutic strategies targeting disulfide stress. This will provide the necessary theoretical foundations and practical guidance for the development of new therapeutic targets and personalized treatment approaches.

### 5.7 Potential therapeutic targets of disulfide stress in CVDs

Disulfide stress is a form of oxidative stress caused by an imbalance in the intracellular thiol-dependent antioxidant system, which manifests as a widespread increase in intracellular disulfides. Therefore, restoring the balance of the intracellular thiol-dependent antioxidant system or reducing the accumulation of peroxides is a promising therapeutic strategy for the treatment of CVDs. Current research focuses mainly on inhibitors of the Trx system, such as PX-12 [111] and APMX 464 [112], which increase disulfide stress in tumor cells, reduce vascular permeability, and promote apoptosis. Additionally, naturally derived bioactive substances, such as puerarin [113,114] and diallyl trisulfide [115,116], can induce the transcription and activation of Trx, thereby reducing intracellular disulfide stress. Synthetic Trx proteins, such as human serum albumin-Trx [117] and xanthine monophosphates [118], play a significant protective role in alleviating arteriosclerosis and I/R injury in mice.

Therapeutic drugs targeting the Grx System are still under development, with many studies using transgenic technology to knock out or overexpress Grx proteins in mouse models to study their effects [119]. Providing reducing equivalents to thiol-dependent antioxidant systems is another promising therapeutic approach. For example, in cancer cells with high SLC7A11 expression, 2-deoxy-d-glucose [120] inhibits the glycolytic pathway, forcing cells to generate NADPH through the pentose phosphate pathway, thus providing sufficient reducing equivalents for the thiol-dependent antioxidant system and reducing the accumulation of disulfide bonds. Additionally, treatments using sulfur-containing compounds, such as N-acetyl cysteine [121] and penicillamine [122], can regenerate free thiol proteins through disulfide exchange reactions, alleviating intracellular disulfide accumulation(Table 1). Studies have shown that such treatments can reduce disulfide stress and apoptosis in renal tumor cells. Overall, disulfide stress holds potential as a therapeutic target in the treatment of CVDs, and more animal experiments and clinical trials are needed to develop more effective medications.

**Table 1:** Potential targets for the application of prevention and treatment of disulfide stress in cardiovascular diseases.

Type	Name	Experimental Cell Models	Mechanism of Action	Outcomes	(Ref)
Trx-1 Inhibitor	PX-12	HT-29, HUVEC, MRCV	Covalently binds to the active site cysteine residue of Trx1, leading to decreased Trx1 activity	Reduces vascular permeability, increases tumor cell apoptosis	[111]
	APMX 464	HT-29, HUVEC, MRCV	Covalently binds to the active site cysteine residue of Trx1, inhibiting Trx1 activity	Decreases tumor cell proliferation with minimal effects on resting cells	[112]
Secondary Metabolites	Puerarin	RAW264.7, ApoE^−/−^ mouse macrophages	Activates PERK/Nrf2 pathway, upregulates Trx1 and TrxR1 expression, reduces intracellular ROS production	Reduces macrophage lipid uptake, decreases lipid deposition in atherosclerotic plaques	[113,114]
	Diallyl Trisulfide (DATS)	U87, U251, MES28, Nude mouse brain tumor model	Directly covalently binds to Trx1's Cys32 and Cys 35 residues, inhibiting Trx1 activity, leading to ROS accumulation	Enhances the cytotoxic effect of radiotherapy on glioma cells, inhibits tumor growth	[115,116]
Recombinant Trx	Recombinant Trx-1	RAW264.7 macrophages	Its active site disulfide bond reduction mechanism, scavenges ROS, inhibits p38 MAPK activation and LOX-1 expression, reducing foam cell formation and apoptosis	Significantly inhibits ox-LDL induced foam cell formation and apoptosis, upregulates Bcl-2 expression, downregulates Bax and caspase-3 expression	[123]
	HSA-Trx	AKI to CKD mice	Its fusion protein's disulfide bond reduction activity, enhances renal antioxidant capacity, inhibits inflammatory response, reduces tubular injury and fibrosis	Accelerates renal function recovery, reduces renal fibrosis, inhibits inflammatory cell infiltration, restores renal tubular cell apoptosis and cell cycle arrest	[117]
	xMPs (CB3)	Murine peritoneal macrophages	Mimics the active site Cys32-Gly-Pro-Cys 35(CXXC) of Trx-1, maintains intracellular redox balance, reduces ROS generation, and diminishes inflammation	CB3 dose-dependently reduces ROS levels in LPS-activated macrophages,	[118]
Direct Disulfide Reduction	2DG Glucose Analogue	UMRC6 cells	Provides NADPH to reduce disulfide bond accumulation	Significantly restores NADPH levels, reduces accumulation of γ-glutamylcysteine and glutathione disulfide, prevents cell death	[120]
	NAC (N-acetylcysteine)	UMRC6 cells	Supplies intracellular cysteine and promotes GSH synthesis	Restores intracellular redox system balance, reduces cell death	[121]
	Penicillamine	UMRC6 cells	Disulfide bond exchange prevents accumulation of cysteine disulfide bonds	Restores intracellular redox system balance, reduces cell death	[122]
Grx-1 Inhibitor	AAV-shGrx	C2C12 skeletal muscle cells	Inhibits Grx-1 expression, inducing disulfide stress	After 6 weeks, Grx mRNA in muscle decreased by 80 %, protein level decreased by 50 %, with localized ROS accumulation	[119]
Grx-1 Overexpression	Adenovirus Grx1	WT and ApoE^−/−^ mice	Reduces glutathionylation of intracellular proteins, inactivates PKB/eNOS, and inhibits Rac1 degradation	Overexpression of Grx in transgenic mice attenuates collagen deposition, inhibits endothelial hypercholesterolemia	[63]

## 6 Concluding remarks and future perspectives

This review discusses the intricacies of disulfide stress, a distinct form of oxidative stress. Oxidative stress plays a pivotal role in the genesis and progression of a myriad of diseases, and involves an imbalance of antioxidant reactions or an increase in ROS that can damage cellular DNA, proteins, and lipids and influence cell death through signal transduction. Disulfide stress arises from the disequilibrium of the intracellular thiol-dependent antioxidant system, leading to a surge in ROS and the formation of aberrant disulfide bonds in proteins, thereby altering their function. Notably, disulfide stress is not solely caused by disequilibrium of the thiol-dependent antioxidant system; excessive external stimuli or experimental conditions can also trigger disulfide stress. In healthy organisms, an imbalance in the thiol-dependent antioxidant system manifests as a decrease in intracellular antioxidant enzyme activity and a weakening of antioxidant capacity, with an increase in ROS that can induce disulfide stress. Furthermore, disulfide stress exacerbates intracellular oxidative stress through various mechanisms, leading to pathological changes such as DNA damage and inhibition of angiogenesis through multiple signaling pathways. Severe disulfide stress can also promote cellular autophagy, apoptosis, and carcinogenesis, thereby altering cellular processes. The Trx and Grx systems are crucial for maintaining the antioxidant balance. Imbalances in the thiol-dependent antioxidant system caused by external factors, such as persistent protein glutathionylation, intermolecular disulfide bond formation, or irreversible oxidation of thiol groups, can induce disulfide stress, leading to ROS accumulation, the collapse of the antioxidant system, and even triggering a novel form of cell death known as “disulfidptosis.” This review also discusses the role of the Trx and Grx system imbalance and the damaging effects of disulfide stress on CVDs, including atherosclerosis, hypertension, myocardial I/R injury, diabetic cardiomyopathy, cardiac hypertrophy, and HF, and proposes potential therapeutic avenues aimed at improving the future treatment of CVDs.

In terms of disulfidptosis, this mode of cell death was discovered in cancer cells with high SLC7A11 expression, in which glucose depletion-induced disulfide stress promotes the formation of disulfide bonds in redox-sensitive proteins, inducing F-actin contraction and detachment from the plasma membrane. This ultimately leads to abnormal disulfide bond formation between actin and cytoskeletal proteins, causing the collapse of the actin network and cell death [121]. This form of death is distinct from traditional forms, such as necroptosis, ferroptosis, and cardioproptosis, and it plays a role in atherosclerosis and myocardial I/R injury. Although the discovery of disulfidptosis did not highlight the role of the antioxidant proteins Grx and Trx, based on subsequent reviews [77,124,125], it is inferred that the cause of this cell death is the reduction in the synthesis of cysteine and NADPH in cells with high expression of SLC7A11 and glucose depletion, affecting the efficacy of antioxidant systems such as the Trx and Grx systems, leading to the accumulation of oxidizing substances within the cell and an increase in disulfides, such as GSSG. Disulfide bonds that should be extensively formed in the endoplasmic reticulum are also formed extensively in the cytoplasm because of stress conditions, and the formation of disulfide bonds in redox-sensitive proteins may be a mechanism to protect proteins from irreversible oxidation and maintain their function. A significant increase in the level of protein glutathionylation in cells under glucose depletion can be detected using non-reducing protein blotting methods [121]. However, continuous external stimuli lead to massive production of disulfide bonds within the cell. When the cell cannot provide sufficient reducing equivalents, it causes abnormal disulfide bond connections between actin and cytoskeletal proteins, leading to the collapse of the cytoskeletal network and induction of disulfidptosis.

Disequilibrium of the thiol-dependent antioxidant system has long been reported to lead to the diminished activity and dysfunction of actin [[126], [127], [128]], highlighting the intimate connection between the thiol-dependent antioxidant system and the stability of cytoskeletal proteins. However, whether the collapse of the cytoskeletal network induced by disulfide stress is the only pathway that induces cell death remains unclear and warrants further investigation. With the advancement of experimental techniques, the redox states of Trx and GSH within living cells can be directly observed to monitor the oxidation-reduction status and intracellular redox potential [129]. As technology progresses, direct observation of the generation, transport, and elimination of intracellular disulfide bonds, along with the potential for human intervention to restore the homeostasis of these bonds, appears to be a feasible therapeutic target for CVDs.

## Funding sources

This research was supported by the Key specialized research and development breakthrough of Henan Province (242102311071), the Central Plains Science and Technology Innovation (244200510003), the Leading Talents and Henan Province Science and Technology Innovation Team Project (23IRTSTHN030), the 10.13039/100018477Startup Foundation for Doctor of 10.13039/501100011841Xinxiang Medical University (XYBSKYZZ202159), and the National Natural Science Foundation of China (82270297)

## CRediT authorship contribution statement

**Shaoju Qian:** Writing – review & editing, Writing – original draft, Data curation. **Guanyu Chen:** Writing – review & editing, Writing – original draft, Visualization. **Ruixue Li:** Writing – review & editing, Writing – original draft, Conceptualization. **Yinghua Ma:** Writing – original draft, Conceptualization. **Lin Pan:** Writing – review & editing, Conceptualization. **Xiaoping Wang:** Writing – review & editing. **Xianwei Wang:** Writing – review & editing, Supervision.

## Declaration of competing interest

The authors declare that they have no known competing financial interests or personal relationships that could have appeared to influence the work reported in this paper.

## Data Availability

No data was used for the research described in the article.
